# PR-LncRNA signature regulates glioma cell activity through expression of SOX factors

**DOI:** 10.1038/s41598-018-30836-5

**Published:** 2018-08-24

**Authors:** Sergio Torres-Bayona, Paula Aldaz, Jaione Auzmendi-Iriarte, Ander Saenz-Antoñanzas, Idoia Garcia, Mariano Arrazola, Daniela Gerovska, Jose Undabeitia, Arrate Querejeta, Larraitz Egaña, Jorge Villanúa, Irune Ruiz, Cristina Sarasqueta, Enrique Urculo, Marcos J. Araúzo-Bravo, Maite Huarte, Nicolas Samprón, Ander Matheu

**Affiliations:** 1 Cellular Oncology group, Biodonostia Institute, San Sebastian, Spain; 2 IKERBASQUE, Basque Foundation for Science, Bilbao, Spain; 3 CIBERFES, Madrid, Spain; 4 Donostia University Hospital, San Sebastian, Spain; 5 Center for Applied Medical Research, University of Navarra, Pamplona, Spain; 6 Computational Biology and Systems Biomedicine group and Computational Biomedicine Data Analysis Platform, Biodonostia Institute, San Sebastian, Spain; 7 Surgery and Radiology Department, School of Medicine, University of the Basque Country UPV/EHU, San Sebastian, Spain; 8 Biodonostia Institute, San Sebastian, REDISSEC, Madrid, Spain

**Keywords:** Long non-coding RNAs, CNS cancer

## Abstract

Long non-coding RNAs (LncRNAs) have emerged as a relevant class of genome regulators involved in a broad range of biological processes and with important roles in tumor initiation and malignant progression. We have previously identified a p53-regulated tumor suppressor signature of LncRNAs (*PR-LncRNAs*) in colorectal cancer. Our aim was to identify the expression and function of this signature in gliomas. We found that the expression of the four *PR-LncRNAs* tested was high in human low-grade glioma samples and diminished with increasing grade of disease, being the lowest in glioblastoma samples. Functional assays demonstrated that *PR-LncRNA* silencing increased glioma cell proliferation and oncosphere formation. Mechanistically, we found an inverse correlation between *PR-LncRNA* expression and *SOX1*, *SOX2* and *SOX9* stem cell factors in human glioma biopsies and in glioma cells *in vitro*. Moreover, knock-down of *SOX* activity abolished the effect of *PR-LncRNA* silencing in glioma cell activity. In conclusion, our results demonstrate that the expression and function of *PR-LncRNA*s are significantly altered in gliomagenesis and that their activity is mediated by SOX factors. These results may provide important insights into the mechanisms responsible for glioblastoma pathogenesis.

## Introduction

Gliomas are relatively rare, around 350,000 people being diagnosed per year worldwide, but they are the most common primary brain tumors and, importantly, account for over 80% of malignant primary central nervous system tumors^1^. They have been classified into different grades of malignancy based on histopathological and clinical criteria, glioblastomas corresponding to the highest grade^2^. Glioblastoma is the most common and malignant primary intracranial tumor in adults, with an incidence ranging from 1 to 5 cases per 100,000 people per year. Current therapy consists of maximal surgical tumor resection followed by concomitant radiotherapy and chemotherapy with temozolomide^2^. This therapy is only partially effective, however, and aggressive growth and recurrence frequently follows even after optimal treatment. In line with this, patients have an associated median survival of 12–15 months and only around 5% of patients survive to 3 years^3,4^. This dismal patient survival identifies glioblastoma as one of the most aggressive and fatal cancers overall. It is therefore necessary to identify strategies and targets for the early diagnosis and therapeutic treatment of gliomas.

Glioblastoma shows significant variability and heterogeneity at clinical, morphological, histopathological, molecular and cellular levels, and this heterogeneity goes a long way to explaining the poor prognosis^2^. Several studies have explored the genetic and molecular characteristics of this type of cancer, providing a high-resolution picture of the glioblastoma landscape, in turn, allowing the identification of different subtypes based on the molecular knowledge of the genome and transcriptome^5–7^. Nevertheless, high-throughput sequencing of whole genomes and transcriptomes found that less than 2% of the genome encodes proteins, whilst 75% is actively transcribed into noncoding RNAs^8^. Increasing evidence is demonstrating that frequent major genomic mutations in cancer reside inside this vast majority of regions that do not encode proteins^9^. These genomic loci are often transcribed into long noncoding RNAs (LncRNAs)^9^.

LncRNAs are transcripts of more than 200 nucleotides that miss functional open reading frames and do not have functional protein-coding ability. Mechanistically, LncRNAs can fold into larger structures to provide higher potential for target recognition, which facilitates chromatin remodeling as well as transcriptional and post-transcriptional regulation^10^. Increasing evidence shows that the levels of LncRNAs are altered in multiple contexts and their deregulation facilitates the modulation of gene expression during both normal biological and pathological processes. Thus, mutations and dysregulations of lncRNAs contribute to the development of several human complex diseases, including cancer^11^. In brain tumors, a microarray analysis identified over 1000 LncRNAs differentially expressed in glioblastoma and healthy brain tissue^12^, with a similar quantity of LncRNAs overexpressed and silenced^12^. Additional studies confirmed differentially expressed LncRNAs in gliomas using further human tissues and also cell lines^13,14^. Notably, these altered LncRNA expression patterns have been correlated with malignancy grade, patient survival and histological differentiation in human gliomas^13,14^. In line with this, the development of effective computational models predicted cancer associated-LncRNAs as biomarkers for glioma diagnosis, treatment and prognosis^15,16^. Moreover, abnormal LncRNA function plays critical roles in the development and progression of gliomas, controlling processes such as proliferation, apoptosis, self-renewal and migration^13,14^. These studies support the hypothesis that LncRNAs assume an important role in glioblastoma pathogenesis. Nevertheless, the exact functions in normal biological and disease processes have been reported for only a few LncRNAs in glioblastoma^17,18^.

Through genome-wide studies, we previously identified a set of LncRNAs, which are differentially expressed upon DNA damage in human colorectal cancer cells in response to active p53^19^. These LncRNAs, called p53-regulated LncRNAs (*PR-LncRNAs*), are required for the efficient binding of p53 to some of its well-known target genes and contribute to p53 pro-apoptotic and cell cycle regulatory functions^19^. Importantly, the expression of the *PR-LncRNA* signature is lower in colorectal cancer samples than healthy adjacent control tissue, suggesting that *PR-LncRNAs* might constitute a tumor-suppressor signature^19^. Since mutations on p53 are common and p53 pathway is frequently deregulated in gliomas^20^, in this work, we characterized the expression and function of the *PR-LncRNA* signature, by studying the expression and clinical relevance of four members of this signature in human glioma samples of different grades and assessing the effect of loss of function in glioma cells.

## Methods and Materials

### Patients and tumor samples

Human glioma patient clinical information were obtained from the Donostia University Hospital. Human glioma samples were provided by the Basque Biobank for Research-OEHUN (http://www.biobancovasco.org). The study included biopsies from 35 patients seen in San Sebastian, and diagnosed as primary glioblastoma grade IV according to the WHO criteria, 4 as a grade III and 4 as a low grade I-II. All study participants signed informed consent form approved by the Institutional Ethical Committee. The study was approved by the ethic committee of Hospital Donostia and all the experiments were performed in accordance with relevant guidelines and regulations.

### RNA analysis

Total RNA was extracted with Trizol (Life Technologies). Reverse transcription was performed using random priming and Maxima First Strand cDNA Synthesis Kit (ThermoFisher), according to the manufacturer’s guidelines. Quantitative real-time polymerase chain reaction (PCR) was performed using Absolute SYBR Green mix (Thermo Scientific) in a CFX384 Real-time thermal cycler (BioRad). Variations in input RNA were compensated for by subtracting the PCR threshold cycle values obtained for *GAPDH*.

### Cell lines and cultures

Glioma cell lines U87-MG (U87), U373MG (U373), U251MG (U251) and A172 purchased from the ATCC, were cultured in DMEM (Gibco) supplemented with 10% FBS (Gibco), 100 U/ml penicillin and 100 µg/ml streptomycin for traditional monolayer cultures. Patient-derived GNS166 and GNS179 cell lines, kindly provided by Dr. Steven Pollard^21^, GB1 established by our group^22^, and oncospheres from glioma cell lines were cultured in DMEM/F-12 (Sigma) supplemented with N2, B27 supplements (Fisher) and growth factors (20 ng/ml basic fibroblast growth factor, and 20 ng/ml epidermal growth factor; Sigma) for oncosphere cultures^22^. Cells were maintained under standard conditions in humidified atmosphere of 5% CO_2_ at 37 °C.

### Transfections with antisense oligonucleotides

The antisense oligonucleotide (ASO) sequences used in this study were designed and provided by ISIS Pharmaceuticals and the methodology has been previously described^19^. Cells were transfected with 50 nM of each ASO, using lipofectamine 2000 (Invitrogen) and inmunofluorescence and mRNA studies were done 48 hours post-transfection.

### Lentiviral infections

Lentiviral infections were performed as previously described^23^. For *SOX* knockdown, cells were infected with *pLKO*.*1 shSOX1* and *pLKO*.*1 shSOX9* or empty vector (obtained from Sigma). Infected cells were selected in the presence of 2 μg/ml puromycin and then maintained with 0.2 μg/ml puromycin (Sigma).

### Oncosphere assays

For quantification studies, 7000 U87-MG cells/well were seeded in non-treated 6-well flat bottom plates and fresh media was added to the plates every 3 days. After 10 days, oncospheres were counted. Then, these oncospheres were disaggregated with accutase (Gibco), and same amount of cells were seeded for secondary oncospheres and maintained in culture for another 10 days.

### Immunofluorescence

Immunofluorescence was performed following standard procedures^24^. The primary antibody was anti-phospho-histone-3 (PH3, 1:2000; Ab14955, Abcam), and the secondary antibody was anti-mouse Alexa Fluor 555 IgG (Invitrogen). Nuclear DNA was stained with Hoechst 33342 (Sigma). Pictures were taken with an Eclipse 80i microscope and processed with the NIS Elements Advances Research software (Nikon).

### Discriminant analysis

The PCR expression of the four *PR-LncRNAs* were used to estimate the heuristic probability distribution functions of LGG and HGG conditions. The heuristic probability distribution functions of LGG and HGG conditions were predicted by fitting the corresponding log_2_ transformed signals using the generalized extreme value (GEV) model. The GEV model parameters: shape *k*, scale, σ and location µ, were estimated using the function fitdis of Matlab (MathWorks^TM^). To estimate the discrimination capability of each *PR-LncRNA*, we calculated the overlap of the LGG and HGG probability distribution functions (PDFs). For each *x* coordinate the overlap is the minumun of between the LGG and HGG PDFs. The *PR-LncRNAs* with best discrimination capabilities are those with smaller overlap between LGG and HGG PDFs. Next, we calculated the performance of all the possible combinations of selected *PR-LncRNA* using performance metrics of the Receiver Operating Characteristic (ROC), namely, the distance to the optimal point (0, 1) of the ROC space (D01), the accuracy, the specificity and the distance to the optimal point (0, 1) of the ROC space (D01) The D01 is calculated as D01 = (1 − TPR^2^ + FPR^2^)^0.5^, were TPR and FPR are the true and false positive rates, respectively. Finally, the combination of *PR-LncRNAs* with smallest D01 and highest accuracy and specificity was selected as optimal predictor. We used the same set of samples for our prospective study to choose the optimal predictor and to make the prediction because of limited number of samples from LGG patients. Data processing and graphics were performed with functions developed in Matlab (MathWorksTM).

## Results

### Expression of PR-LncRNA signature decreases with glioma grade

**Figure 1:**
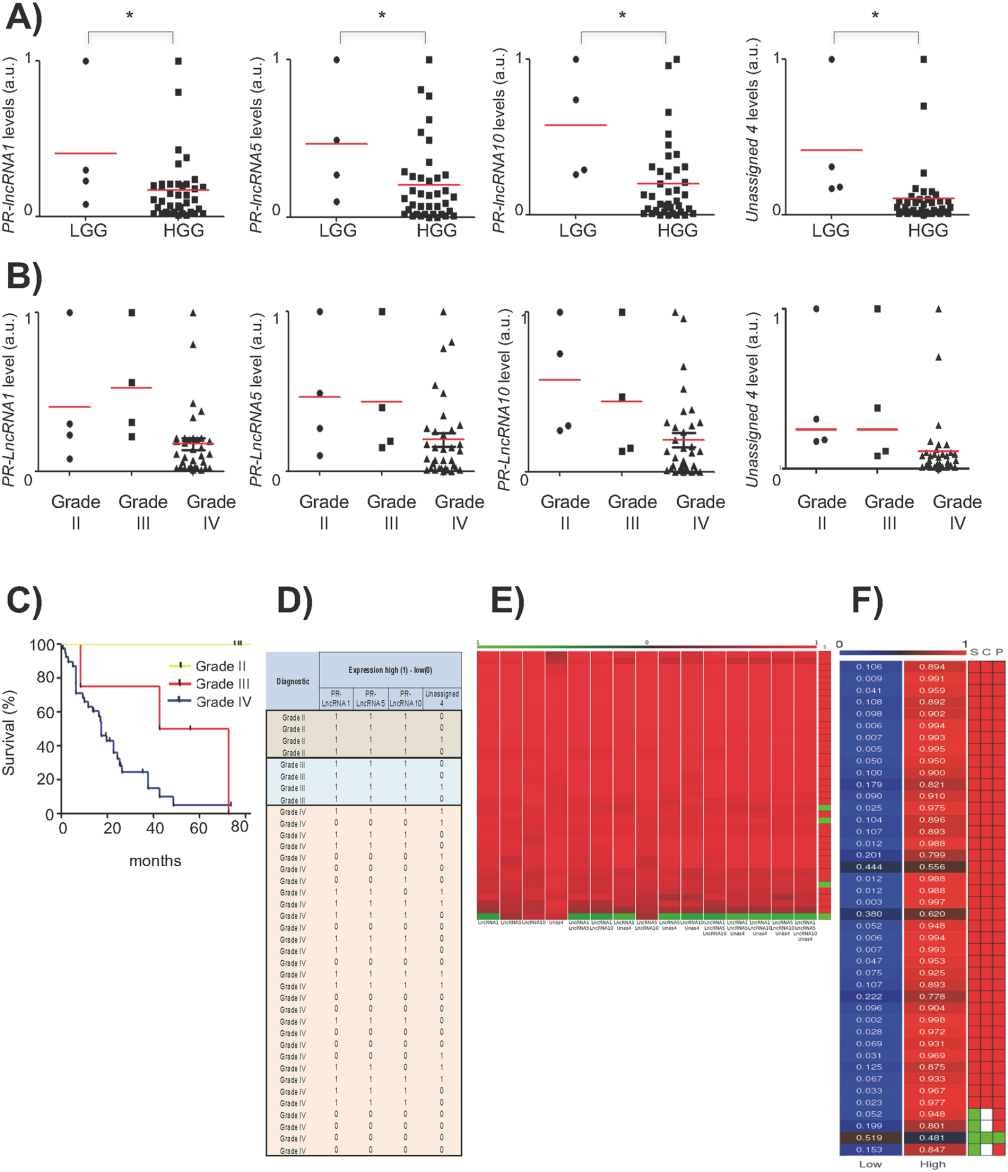
PR-LncRNA expression in increasing glioma grade samples. (**A**) Comparison of *PR-LncRNA 1*, *5*, *10* and *Unassigned 4* expression in low (grade II) and high-grade (III and IV) glioma (LGG and HGG respectively). (**B**) Expression of *PR-LncRNA 1*, *5*, *10* and *Unassigned 4* expression in grade II, III and IV gliomas. (**C**) Kaplan-Meier curve representing the survival of patients with grade II (n = 4), grade III (n = 4) and grade IV (glioblastoma) tumors (n = 35). (**D**) List of glioma biopsies with high (1) or low(0) (above or below median) expression of *PR-LncRNA 1*, *5*, *10* and *Unassigned* 4 (**E**) Heat map of the prediction of a sample to be HGG or LGG using all different combinations (in columns) of expression of *PR-LncRNAs* as predictors. The colorbar on the top shows the probability of a sample to be HGG or LGG. The negative values are associated with the probabilities of LGG and the positive with the probabilities of HGG. Each row represents the probabilities for a patient. The column table to the right shows the real status of the sample: red for HGG and green for LGG. The optimal predictor variable combination for the discrimination analysis between LGG and HGG grade glioma is chosen as the one with lowest value of the Receiver Operating Characteristic (ROC) of the performance of the predictions and highest value of Accuracy, Specificity and Sensitivity, namely *PR-LncRNA 1*. (**F**) Heat map of the predictive value of a sample to be HGG (High) or LGG (Low) using the expression of *PR-LncRNA 1* as the optimal predictor. The colorbar on the top shows the probability of a sample to be HGG. The table to the right of the heatmap shows: S, real status of the sample; P, predicted status of the sample; C, comparison between the real and the predicted status. Red color is used for HGG, green for LGG, and white when there is no coincidence between the real and the predicted status using this optimal predictor, *PR-LncRNA 1*Asterisks (*) indicate statistical significance (p < 0.05).

To characterize the expression of the *PR-LncRNAs* in human glioma samples, we measured the expression of four of the *PR-LncRNAs* (*PR-LncRNAs* 1, 5 and 10 and Unassigned 4) in a set of biopsies including 4 low-grade gliomas (grade II), 4 anaplasic gliomas (grade III) and 35 glioblastomas (grade IV) obtained from a cohort of patients from Donostia Hospital. First, we found that the levels of the four tested *PR-lncRNAs* were significantly higher in low-grade than high-grade gliomas (Fig. 1A). The analysis across the different tumor grades revealed that *PR-lncRNA* expression gradually decreased with the advance of glioma malignancy with the exception of *PR-lncRNA-*1 (Fig. 1B). In line with previous studies^2^, patient survival in the Donostia Hospital cohort decreased with advancing glioma grade (Fig. 1C), with a median survival of 77, 49 and 17 months in grades II, III and IV respectively.

Next, we studied whether the expression of *PR-LncRNAs* could form a signature in glioma samples and analyzed their putative association. Indeed, there were significant positive correlations between the expressions of the different *PR-LncRNAs* (p < 0.001) (Fig. 1D). Moreover, we developed a discriminant analysis method to study the predictive capabilities of the *PR-LncRNA* to predict low-grade or high grade gliomas. We found that the optimal combination of *PR-LncRNAs* is actually formed by a single *PR-LncRNA*, namely *PR-LncRNA 1* (Fig. 1E). Finally, we used the optimal predictor *PR-LncRNA 1* to perform the discriminant analysis on our sample set. We found that the optimal discriminator classified correctly 93% of the samples, with 100% for the case of high-grade glioma, and 25% for low grade (Fig. 1F). The low prediction rate of low-grade glioma is due to the lack of higher number of available patients (n = 4). These results show that *PR-LncRN*A levels do represent a signature, expression diminishing with glioma grade.

### PR-LncRNA levels in glioma samples correlate with clinical characteristics

We performed correlation analysis between the expression of the *PR-LncRNAs* and clinical data concerning Donostia University Hospital glioma cohort. We first analyzed the clinical data for the cohort. Patients were distributed homogeneously in terms of sex, and overall survival did not vary by sex (Table 1). Most of the patients were under 65 years old and, notably, the risk of death was 3.5-fold higher in those who were ≥65 of years age (HR = 1.046, CI 1.008–1.085) (Table 1). The majority of patients were autonomous (Karnofsky score ≥70) at the time of diagnosis, but a preoperative score <70 correlated with poorer outcome (P < 0.007) (Table 1). Finally, patients who underwent complete surgical resection of the tumor (67%) survived for longer than those with subtotal extirpation (p = 0.022) (Table 1). On the other hand, we did not observe any significant correlation between patient survival and molecular markers such as MGMT, IDH1, ATRX, EGFR and p53 status (Table 2)

**Table 1:** Clinical and radiological characteristics of the glioma cohort from Donostia Hospital.

Clinical/pathological characteristics	Number of patients	Low grade (I-II)	Anaplasic (III)	Glioblastoma (IV)	P Value Survival
**Glioma Grade**	43	4	4	35	0.0031
**Sex**	43				
Male		2	2	17	NS
Female		2	2	18	
**Age, years**	43				
≥65		4	2	7	
<65		0	2	28	
**Localization**	43				
Frontal		1	2	16	NS
Temporal			1	13	NS
Parietal			1	2	NS
Other		3		4	NSNS
Multifoci				5	
**Right**	43	2	4	25	0.000***
**Left**				13	NS
**Bilateral**		2		5	NS
**Eloquent**	43	1	1	12	0.001***
**Ventricular contact**	43	2	3	20	**NS**
**Radiological Characteristics**					
Tumor Volume					
≥50	43				−0.069
<50		1	3	20	
Perfusion		3	1	15	
≥6	39	0	0	24	−0.706
<6		0	4	11	
**Karnofsky score**	43				
≥70		3	4	28	0.007**
<70		1	0	7	
**Resection**	43				
Total		4	3	22	0.022*
Subtotal		0	1	13

**Table 2:** Molecular characteristics of the glioblastoma cohort from Donostia hospital.

Molecular characteristics	TOTAL	Low grade (I-II)	Anaplasic (III)	GBM (IV)	P Value vs Survival
**Genes**					
P53 expression	38	1/1	**3/3**	**23/34**	NS
High EGFR	35	ND	**ND**	**22/35**	NS
Mutated IDH1	33	ND	**2/4**	**0/29**	NS
ATRX loss	18	ND	**ND**	**13/18**	NS
MGMT methylation	11	1/1	**3/4**	**3/6**	NS
Ki67 index≥4	40	0/4	**3/3**	**33/33**	NS

**Figure 2:**
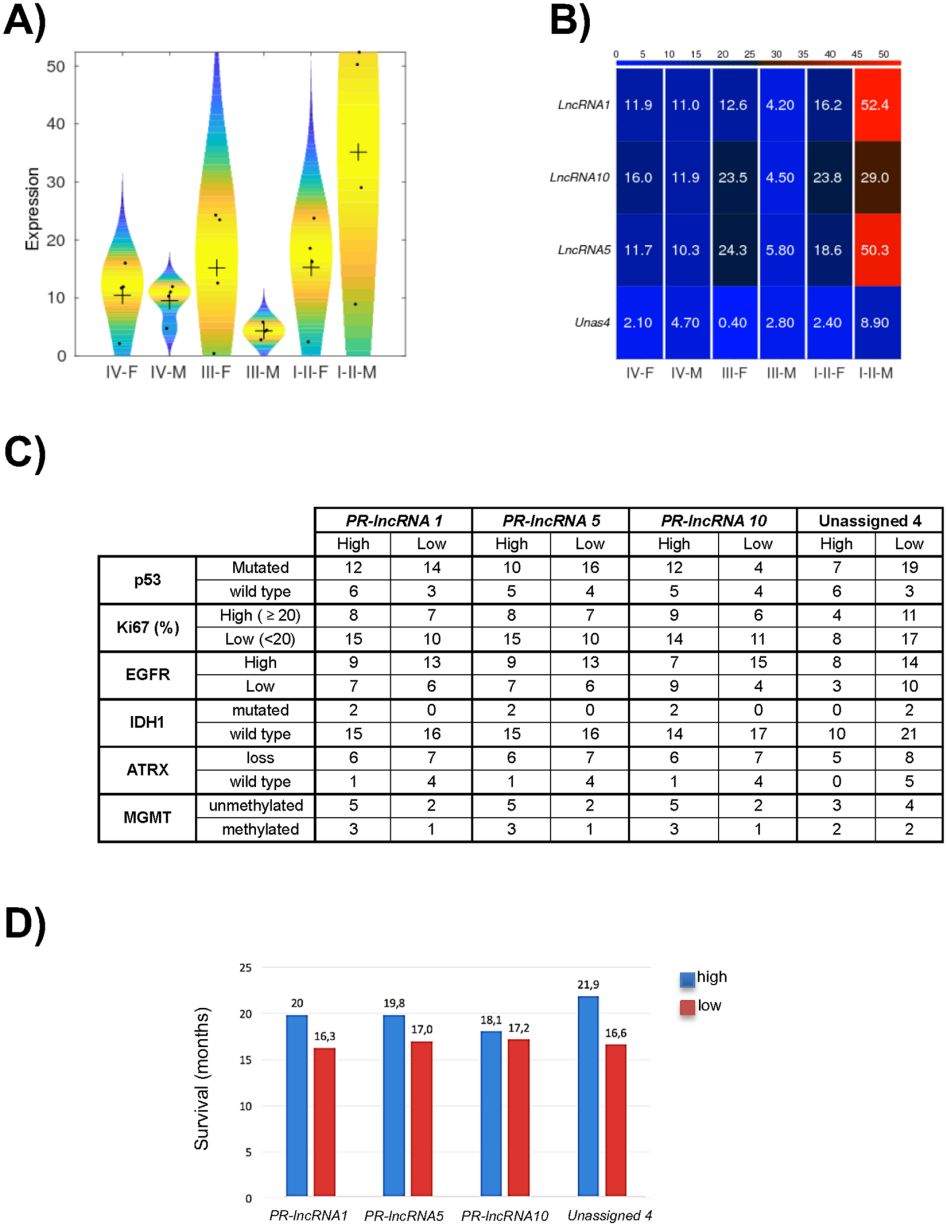
Correlation between PR-LncRNA expression and patient clinical characteristics. (**A**) Violin plot associated to the distribution of the expression of the *PR-LncRNAs* in gliobastoma (IV), anaplastic (III) and low-grade glioma (II) separated by sex (M - male, F - female). The + denote the mean values of the distributions and the black dots are the expression values. (**B**) Heat map of the expression of the studied *PR-LncRNAs* separated by sex. Redder color corresponds to higher expression. The colorbar on the top codifies the expression level. (**C**) Correlation between expression of *PR-LncRNAs* and molecular markers frequently altered in glioma, namely, p53, Ki67, EGFR, IDH1, ATRX and MGMT. (**D**) Median overall survival stratified by level of each of the *PR-LncRNAs* studied.

Next, we compared the expression of *PR-LncRNAs* with the aforementioned variables, finding that the means of the expression of the four *PR-lncRNAs* decreased from grade II to grade IV in female patients, and also in male with the exception of grade III (Fig. 2A). The trend of gradual decrease from lower to higher grade glioma is observed particularly in the expression of *PR-LncRNA1* and 10 in female patients (Fig. 2B). Interestingly, for *PR-LncRNA1*, 5 and 10, the expression for female patients is higher than for male patients in grade III and glioblastoma (Fig. 2B, Table 3). These results indicate that *PR-LncRNAs* expression is sex related. On the contrary, we did not find significant differences in expression of *PR-LncRNAs* by age or pre-operative Karnofsky score (Table 3). Regarding tumor location, more than half of the tumors with high *PR-LncRNAs* were in frontal or temporal locations, the right hemisphere and a non-eloquent area (Table 3). Similar percentages had tumor volumes greater than 50 mm^3^ and a magnetic resonance imaging perfusion parameter higher than 6 (Table 3). When comparing the expression of *PR-LncRNAs* with MGMT methylation status, and Ki67, IDH1, ATRX and EGFR expression, we did not detect any significant associations (Fig. 2C). We observed that majority of samples with high levels of *PR-LncRNAs* had *wild type* p53, while over 50% of cases with low expression of *PR-LncRNAs* had mutated p53 genes, although this difference was not statistically significant (Fig. 2C).

**Table 3:** Correlation between *PR-LncRNA* and clinical characteristics in the glioma cohort.

Clinical/pathological characteristics	Total cases	#	*PR-LncRNA* Expression								
			*PR-LncRNA 1*			*PR-LncRNA 5*			*PR-LncRNA 10*		
			High	Low	P Value	High	Low	P Value	High	Low	P Value
**Sex**											
Male	43	22	15	6	ns	13	8	0.05*	14	7	ns
Female		21	20	2		19	3		19	3	
**Age, years**											
>65	43	13	11	2	ns	10	3	ns	10	3	ns
<65		30	24	6		21	9		23	7	
**Localization**											
Frontal		20	14	6	ns	12	8	0.04*	20	8	0.019*
Temporal		18	17	1	ns	16	2	ns	17	1	0.021*
Parietal		6	4	2	ns	3	3	ns	4	2	ns
Other		10	10	0	ns	10	0	0.034*	10	0	0.048*
Multifoci		5	4	1	ns	4	1	ns	5	0	ns
Right		23	19	4	ns	16	7	ns	17	6	ns
Left		17	14	3	ns	14	3	ns	14	3	ns
Bilateral		3	2	1	ns	2	1	ns	2	1	ns
Eloquent		11	9	2	ns	8	3	ns	8	3	ns
Ventricular contact		18	16	2	ns	16	2	0.065*	16	2	ns
**Radiological Characteristics**											
Volume											
>50	43	24	19	5	ns	17	7	ns	17	7	ns
<50		19	16	3		15	4		16	3	
Perfusion											
>6	39	24	19	5	ns	17	7	ns	17	7	ns
<6		15	12	3		11	4		12	3	
**Karnofsky score**											
>70	43	35	27	8	ns	24	11	ns	26	9	ns
<70		8	8	0		8	0		7	1	
**Resection**											
Total	43	29	23	6	ns	21	8	ns	22	7	ns
Subtotal		14	12	2		11	3		11	3	

Finally, we compared *PR-LncRNA* expression with overall glioblastoma patient survival and we did not observe significant correlations, although we noticed that patients with high levels had longer overall survival for all *PR-LncRNAs* (Fig. 2D). The mean survival rates of the subgroup of patients with high levels of *PR-LncRNAs 1*, *5* and 10, and Unassigned 4 were 20, 19.8, 18.1 and 21.9 months respectively, and notably, the survival rates were lower when biopsies presented low levels of these *PR-LncRNAs*: 16.3, 17, 17.2 and 16.6 months respectively (Fig. 2D). These data indicate a 2–4 month longer survival in patients with higher *PR-LncRNA* levels. In summary, these results show that the expression of *PR-LncRNAs* is altered in glioblastoma samples and low levels of these ncRNAs tend to correlate with characteristics linked to increased malignancy.

### Silencing of PR-LncRNAs increases cellular proliferation and stem cell properties

**Figure 3:**
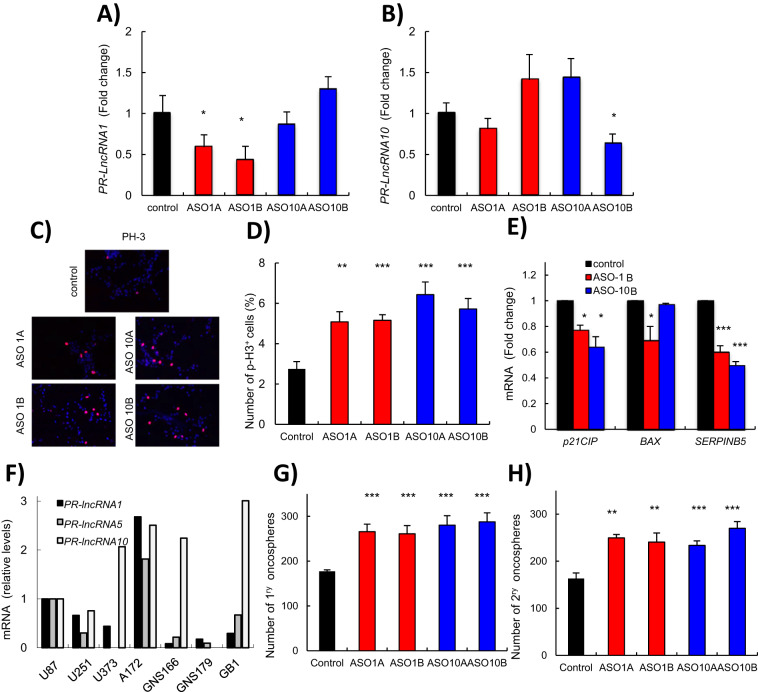
*PR-LncRNA1* and *10* silencing leads to increased proliferation and stemness. U87-MG cells were transfected with specific ASOs for the *PR-LncRNAs* indicated. (**A,B**) Transfected cells were examined for *PR-LncRNA1* and *PR-LncRNA10* expression by quantitative reverse transcription polymerase chain reaction (n = 4). (**C**) Representative immunofluorescence of P-H3 in U87MG cells under the conditions indicated. (**D**) Quantification of the number of P-H3 positive cells under the conditions indicated (n = 4). (**E**) Quantification of mRNA levels of *p21*^*cip*^, *Bax and SerpinB5* in cells transfected with ASOs for *PR-LncRNA1 and 10* and compared to cells with a control ASO (**F**) Expression of *PR-LncRNA 1*,*5* and 10 in indicated conventional cell lines (U87-MG, U251, U373 and A172) and glioma stem cells (GNS166, GNS179 and GB1) (**G**) Quantification of primary oncospheres formed in ASO-transfected cells after 10 days in culture (n = 3). **(H)** Quantification of number of secondary oncospheres generated from disaggregating primary oncospheres in ASO-transfected and control cells. Numbers were assessed after 10 days in culture (n = 3). Asterisks (*^,^**and ***) indicate statistical significance (p < 0.05, p < 0.01, and p < 0.001, respectively).

In order to characterize the impact of *PR-LncRNAs* at the cellular level, we inhibited the activity of *PR-LncRNAs* by transfecting two specific ASOs for *PR-LncRNA1* and 10 respectively. Quantitative reverse transcription PCR showed a significant decrease in the expression of the specific *PR-LncRNA* (Fig. 3A,B). Importantly, when we compared the proliferative potential of control and *PR-LncRNA-*silenced glioma cells, we detected that the number of phospho-histone-3 (PH-3) positive cells was markedly higher in cells transfected with the ASOs for *PR-LncRNA1* and 10 (Fig. 3C,D). Indeed, 3.05% of controls were positive for PH3 compared to 4.9 and 5.38 for *PR-LncRNA1* ASOs and 6.84 and 6.1 for *PR-LncRNA10* ASOs. Moreover, the expression of p53 downstream targets such as *p21*^*cip*^, *BAX* and *SerpinB5* was lower in cells transfected with ASOs for *PR-LncRNA1* and 10 (Fig. 3E). These results support at molecular level the anti-proliferative activity of *PR-LncRNAs* and, since U87-MG cells present p53 wild-type status, the link with p53 pathway.

Glioma stem cells (GSCs) are a subpopulation of cells linked to glioma malignancy, progression and postulated as therapeutic targets^25^. We observed that the expression of *PR-LncRNAs*, particularly *PR-LncRNA1 and 5*, was lower in patient derived GSC lines (GNS166, GNS179 or GB1) than in conventional glioma cell lines such as U87MG, U251 or A172 (Fig. 3F). Moreover, the ability to form oncospheres was significantly elevated in cells with silencing of *PR-LncRNA1* and 10 expression (Fig. 3G). This idea was further corroborated as cells with *PR-LncRNA1* and 10 silencing formed significantly more secondary oncospheres (Fig. 3H). In summary, these results demonstrate that *PR-LncRNAs* regulate the activity of glioma cells.

### SOX factors mediate PR-LncRNAs activity

**Figure 4:**
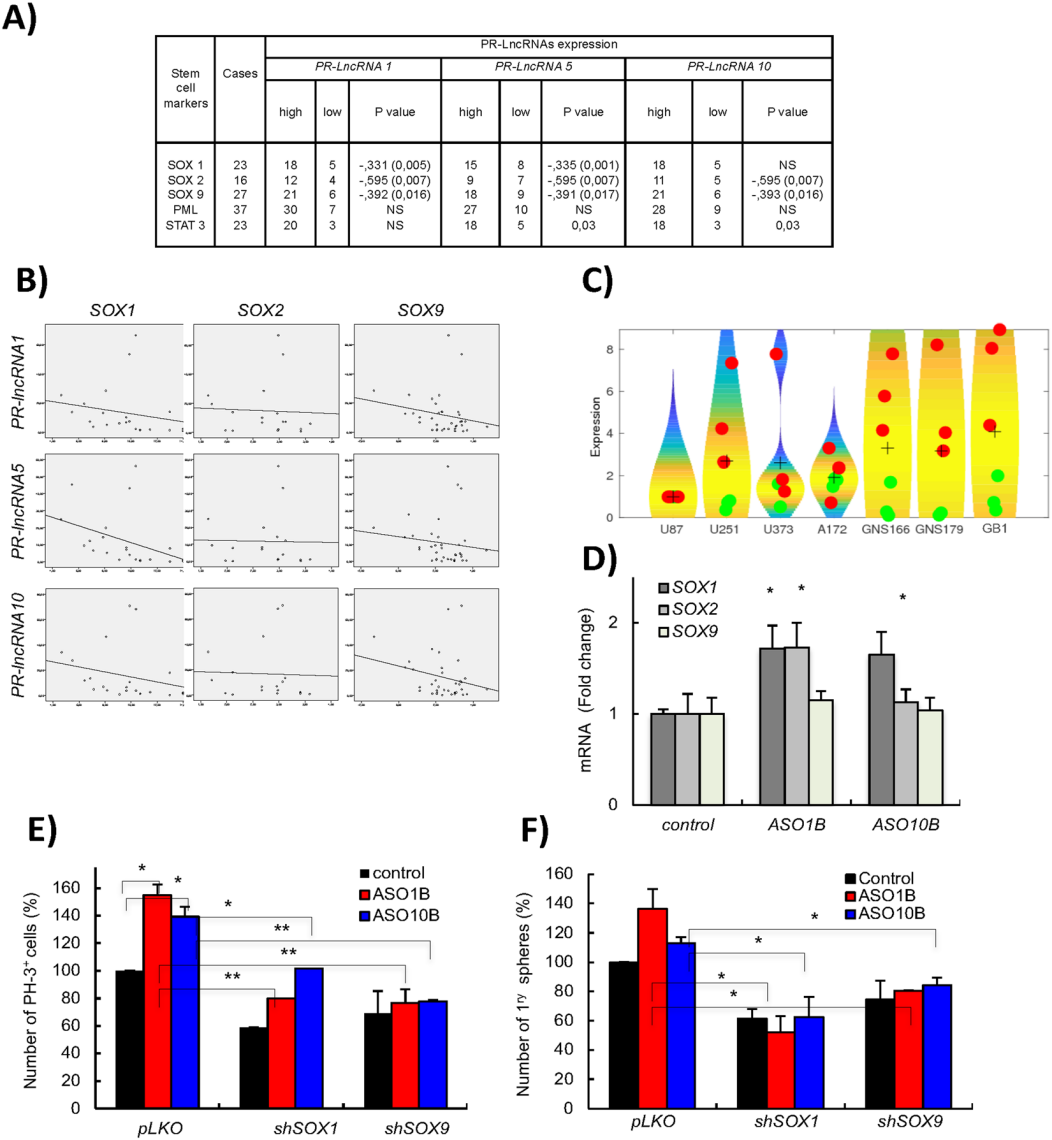
*SOX* stem cell factors mediate activity of *PR-LncRNAs*. (**A**) Analysis of the association between expression of *PR-LncRNAs* and stem cell factors in human glioma samples, showing there is a significant inverse correlation between *PR-LncRNAs* and *SOX* members. (**B**) Correlation between *PR-LncRNAs* and *SOX* factors in human glioma samples. Spearman Correlation Rho is −0,546 (**), −0,221 and −0,342 (*) for *SOX1*, *SOX2* and *SOX9* with *PR-LncRNA1*. It is −0,670 (***), −0,151 and −0,285 (*) for *SOX1*, *SOX2* and *SOX9* with *PR-LncRNA5*. Finally numbers are −0,521 (**), −0,171 and −0,230 for *SOX1*, *SOX2* and *SOX9* with *PR-LncRNA10*. (**C**) Violin plots associated to the expression distribution of the *PR-LncRNAs* (green circles) and *SOX* factors (red circles). Values in indicated cells are relative to expression of each transcript in U87-MG cells. The + denote the mean values of the distributions and the black dots are the expression values. (**D**) Quantitative reverse transcription polymerase chain reaction of *SOX1*, *SOX2* and *SOX9* in U87-MG control and transfected with ASOs. Data represents the average of 3 independent experiments. (**E**) Quantification of the number of P-H3 positive cells in U87-MG cells transfected with indicated ASOs and lentivirally infected with *shSOX1*, *shSOX9* or empty vector (*pLKO*) (n = 2). (**F**) Quantification of the number of formed oncospheres in U87 cells transfected with indicated ASOs and lentivirally infected with *shSOX1*, *shSOX9* or empty vector (*pLKO*) (n = 2). *P* values were determined by Student’s *t* test. Asterisks (*^,^**) indicate statistical significance (p < 0.05 and p < 0.01).

We and others have previously described several regulators of GSCs activity such as members of the *SOX* family, PML and STAT3^22,24–29^. Therefore, we studied whether they were related to *PR-LncRNAs* activity. Correlation analysis in human biopsies determined that there is a strong inverse correlation between expression of the different *PR-LncRNAs* and that of *SOX1*, *SOX2* and *SOX9* (Fig. 4A,B). The inverse association in human biopsies was especially strong for *SOX1* and *SOX9* (Fig. 4B). Similar correlation was observed in glioma cells and GSCs, the latter expressing high levels of SOX members (Fig. 4C). To further investigate the link between *PR-LncRNAs* and *SOX* members, we studied the expression of the latter in cells transfected with *PR-LncRNA1* and 10 ASOs. Interestingly, we observed that specially *SOX1* and *SOX2* expression was elevated in those cells (Fig. 4D), suggesting that these members of the *SOX* family may be mediators of *PR-LncRNA* activity in gliomas. On the contrary, cells with overexpression and silencing of *SOX1*, *SOX2* and *SOX9* do not present a clear pattern of decreased and increased levels of *PR-LncRNAs* respectively (Fig. Suppl 1). These results indicate that *PR-LncRNA* act upstream SOX family members to regulate glioma cell activity. To further test this idea, *SOX1* and *SOX9* expression was knocked-down in cells transfected with ASOs for *PR-LncRNA1* and 10. Interestingly, silencing of *SOX1* or *SOX9* abolished the increase in proliferation and oncosphere formation promoted by *PR-LncRNA1* and *10* inhibition (Fig. 4E,F), experimentally demonstrating that SOX factors are critical mediators of *PR-LncRNA* activity.

## Discussion

LncRNAs are a novel group of genome regulators for which there is growing evidence linking their altered expression to a variety of cancers. In this work, we studied the expression and function of a recently described *PR-LncRNA* signature^19^ in gliomas. We found that the expression of this set of *PR-LncRNAs* is altered in samples from different glioma subtypes. Specifically, their levels are higher in low-grade than in high-grade gliomas, and decrease as the grade increases, with the lowest levels observed in glioblastoma samples. These results are in agreement with the previous study in which this set of *PR-LncRNAs* was found to be downregulated in colorectal cancer samples compared to levels in healthy control tissue^19^.

Clinically, we observed that patients with low levels of *PR-LncRNAs* had tumors in frontal or temporal sites, the right hemisphere and areas in contact with ventricles, characteristics, which have been associated with shorter patient survival^30,31^. In line with this, the subset of biopsies with low levels of the *PR-LncRNAs* studied overlapped with the subgroup of patients with the lowest median overall survival, although the association was not significant, likely due to the small size of the cohort. Building on the association of downregulated levels of *PR-LncRNAs* with poor clinical prognosis, in the previous study in colon cancer^19^, our findings indicate that the signature of *PR-LncRNAs* is not exclusive to human colorectal cancer and suggest that the pattern of expression of these *Lnc-RNAs* might constitute a tumor suppressor signature in different types of cancer. This activity might be influenced by the gender since female patients present higher levels of *PR-LncRNAs* particularly in grade III and glioblastomas. These results further support the link of *PR-LncRNAs* and poor prognosis because there is evidence that female patients have a survival advantage and live longer than males in glioblastoma^32^.

There is an increasing interest among scientists to generate tools to predict clinical phenotypes and tumor progression in order to identify biomarkers and to better design patient treatment^33^. Indeed, there have been generated computational models for lncRNA-cancer association prediction^11,34^. In our study, we generated computational models in order to determine whether the expression of *PR-LncRNAs* could predict glioma grade progression. However, the *PR-LncRNA* signature only discriminated partially between low and high-grade gliomas due to the very low amount of low-grade cases.

Deregulation of the p53 pathway is well known to occur and play a major role in the development and progression of several types of cancer including glioblastoma^20^. The *PR-LncRNA* signature was originally identified as p53-regulated *LncRNAs*^19^. We did not detect a statistically significant association between p53 status and PR-*LncRNA* expression, although most of the biopsies with high levels of PR-*LncRNAs* were positive for *wild-type* p53 in immunohistochemical analysis, and several p53 downstream targets were decreased in U87-MG cells with PR-*LncRNAs* silencing. It is important to indicate that the *PR-LncRNA* signature was identified as p53 regulated upon DNA damage in the HCT116 colorectal cancer cell line^19^. It is reasonable to surmise that: (i) the response of the *PR-LncRNAs* might be different in clinical samples and cell lines and also upon stressful conditions *in vitro*; (ii) the activity and regulation of the signature might be partially different within the different types of cancer; and (iii) some of the *PR-LncRNAs* might be regulated in a p53-independent manner, given that only around ~3% of the *PR-LncRNAs* altered by DNA damage in colorectal cells were directly bound by p53^19^. Further, the association study between p53 protein levels and *PR-LncRNAs* in human colorectal cancer biopsies has yet to be established. Therefore, further studies need to be conducted with a larger number of samples and in different types of cancer to decipher the link between p53 and *PR-LncRNAs* in clinical samples. Independently of all these possibilities, our observations confirm the previous results and link expression of the *PR-LncRNA* signature with poor prognosis and increased malignancy in clinical samples.

In agreement with the results obtained in clinical practice and in colorectal cancer cells^19^, functional studies in glioma cells demonstrated that *PR-LncRNA* silencing increased the proliferative capacity of cells supporting the potential role of *PR-lncRNAs* as a tumor suppressor signature in glioma. This effect was regulated, at least in part, by p53 pathway and SOX members. Importantly, we also observed that *PR-LncRNAs* regulate the activity of glioma stem cells. Indeed, silencing of the *PR-LncRNAs1 and* 10 significantly increased the oncosphere formation ability of glioma cells and their self-renewal potential. Moreover, the levels of *PR-LncRNAs* were lower in patient derived glioma stem cell populations than in differentiated conventional glioma cell lines. Of note, there is a small proportion of *LncRNAs* that have been shown to regulate the activity of the subpopulation of glioma stem cells^13,35,36^, and little is known concerning the underlying molecular mechanisms regulating this activity^37^. Therefore, we tried to identify the downstream mechanism of this action revealing that *PR-LncRNAs* regulate the expression of *SOX2 SOX9* and especially *SOX1*, members of the SOX family transcription factor and stem cell regulators^38^. It is noteworthy that high levels of these genes have been previously linked to glioma poor prognosis and overall shorter survival in clinical practice and glioma stem cell population at the cellular level^22,24,27,39^. Thus, we observed that expression of *PR-LncRNA* was significantly inversely correlated with that of *SOX2*, *SOX9* and *SOX1* in human clinical biopsies and in cell cultures *in vitro*. Moreover, experiments *in vitro* revealed that their levels, especially of *SOX1* and *SOX2*, were significantly elevated in *PR-LncRNA* silencing cells and that knock-down of *SOX1* and *SOX9* expression dramatically reduced the pro-oncogenic activities promoted by *PR-LncRNA* silencing in glioma cells. Together, these results postulate the *SOX* family as novel and critical mediator of the *PR-LncRNAs* activity. In line with these results, gain and loss of function studies demonstrated that SOX members regulate glioma stem cell function as well as glioma cell proliferation and tumorigenic activity^27^.

It has been postulated that understanding the function and mechanism by which LncRNAs participate in glioma stem cell activity might facilitate development of therapeutic strategies for the treatment of the disease^14^. In summary, our study identified a novel LncRNA signature associated with glioma progression and malignancy in the clinic and revealed the tumor suppressor role played by these LncRNAs at cellular level. Our data also revealed that members of the *SOX* family of stem cell regulators, in particular the recently described *SOX1*^24^, are critical mediators of their activity. These results unravel the expression and function of the PR-LncRNAs in glioma pathobiology, postulating them as novel biomarkers in glioma diagnosis, especially for female patients, as well as potential therapeutic targets.

## Electronic supplementary material

Supplementary figures
