# Author Correction: PR-LncRNA signature regulates glioma cell activity through expression of SOX factors

**DOI:** 10.1038/s41598-022-13824-8

**Published:** 2022-06-20

**Authors:** Sergio Torres-Bayona, Paula Aldaz, Jaione Auzmendi-Iriarte, Ander Saenz-Antoñanzas, Idoia Garcia, Mariano Arrazola, Daniela Gerovska, Jose Undabeitia, Arrate Querejeta, Larraitz Egaña, Jorge Villanúa, Irune Ruiz, Cristina Sarasqueta, Enrique Urculo, Marcos J. Araúzo-Bravo, Maite Huarte, Nicolas Samprón, Ander Matheu

**Affiliations:** 1 Cellular Oncology Group, Biodonostia Institute, San Sebastian, Spain; 2 IKERBASQUE, Basque Foundation for Science, Bilbao, Spain; 3 CIBERFES, Madrid, Spain; 4 Donostia University Hospital, San Sebastian, Spain; 5 Center for Applied Medical Research, University of Navarra, Pamplona, Spain; 6 Computational Biology and Systems Biomedicine Group and Computational Biomedicine Data Analysis Platform, Biodonostia Institute, San Sebastian, Spain; 7 Surgery and Radiology Department, School of Medicine, University of the Basque Country UPV/EHU, San Sebastian, Spain; 8 Biodonostia Institute, San Sebastian, REDISSEC, Madrid, Spain

Correction to: *Scientific Reports* 10.1038/s41598-018-30836-5, published online 24 August 2018

The original version of this Article contained errors in Figure 3.

In Figure 3C, the representative image for ASO10A was inadvertently duplicated as ASO1B. Data from the fourth experimental replicate was also omitted from Figure 3D. The original Figure 3 and accompanying legend appear below.

**Figure 3:**
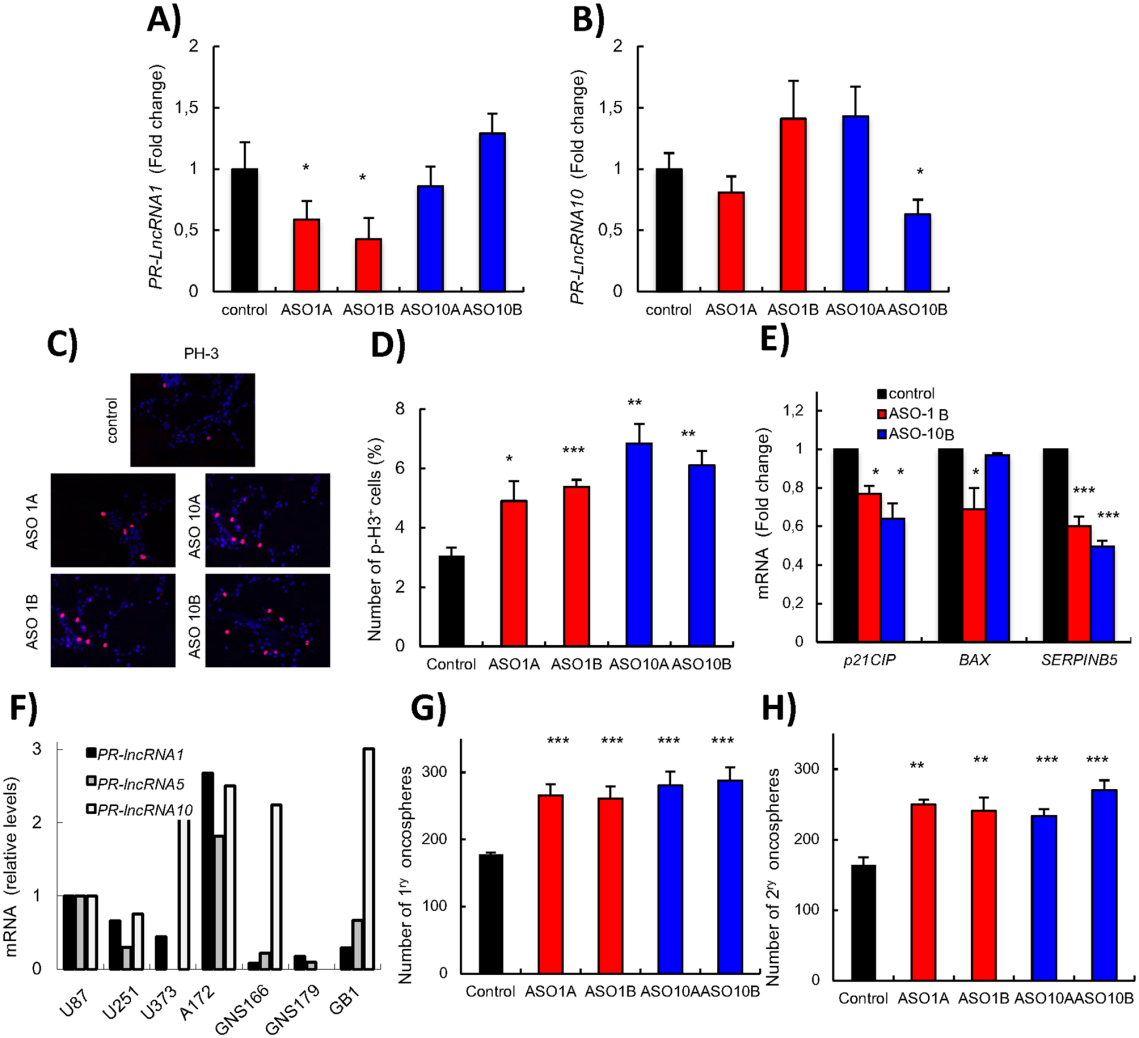
*PR-LncRNA1* and *10* silencing leads to increased proliferation and stemness. U87-MG cells were transfected with specific ASOs for the *PR-LncRNAs* indicated. (**A,B**) Transfected cells were examined for *PR-LncRNA1* and *PR-LncRNA10* expression by quantitative reverse transcription polymerase chain reaction (n = 4). (**C**) Representative immunofluorescence of P-H3 in U87MG cells under the conditions indicated. (**D**) Quantification of the number of P-H3 positive cells under the conditions indicated (n = 4). (**E**) Quantification of mRNA levels of *p21*^*cip*^, *Bax and SerpinB5* in cells transfected with ASOs for *PR-LncRNA1 and 10* and compared to cells with a control ASO (**F**) Expression of *PR-LncRNA 1,5* and 10 in indicated conventional cell lines (U87-MG, U251, U373 and A172) and glioma stem cells (GNS166, GNS179 and GB1) (**G**) Quantification of primary oncospheres formed in ASO-transfected cells after 10 days in culture (n = 3). (**H**) Quantification of number of secondary oncospheres generated from disaggregating primary oncospheres in ASO-transfected and control cells. Numbers were assessed after 10 days in culture (n = 3). Asterisks (*,**and ***) indicate statistical significance (*p* < 0.05, *p* < 0.01, and *p* < 0.001, respectively).

The original Article has been corrected.

