# Supplementary material for: PR-LncRNA signature regulates glioma cell activity through expression of SOX factors
Source: Sci Rep. 2018 Aug 24;8:12746. doi: 10.1038/s41598-018-30836-5 (PMC6109087; doi:10.1038/s41598-018-30836-5)
Supplement: Supplementary file 1 — Supplementary figures [file 41598_2018_30836_MOESM1_ESM.pdf]

SUPPLEMENTARY INFORMATION

PR-LncRNA signature regulates glioma cell activity through expression of SOX factors

Sergio Torres-Bayona, Paula Aldaz, Jaione Auzmendi-Iriarte, Ander Saenz-Antoñanzas, Idoia Garcia, Mariano Arrazola, Daniela Gerovska, Jose Undabeitia, Arrate Querejeta, Larraitz Egaña, Jorge Villanúa, Irune Ruiz, Cristina Sarasqueta, Enrique Urculo, Marcos Arauzo-Bravo, Maite Huarte, Nicolas Samprón , Ander Matheu.

Manuscript SREP-18-10537A

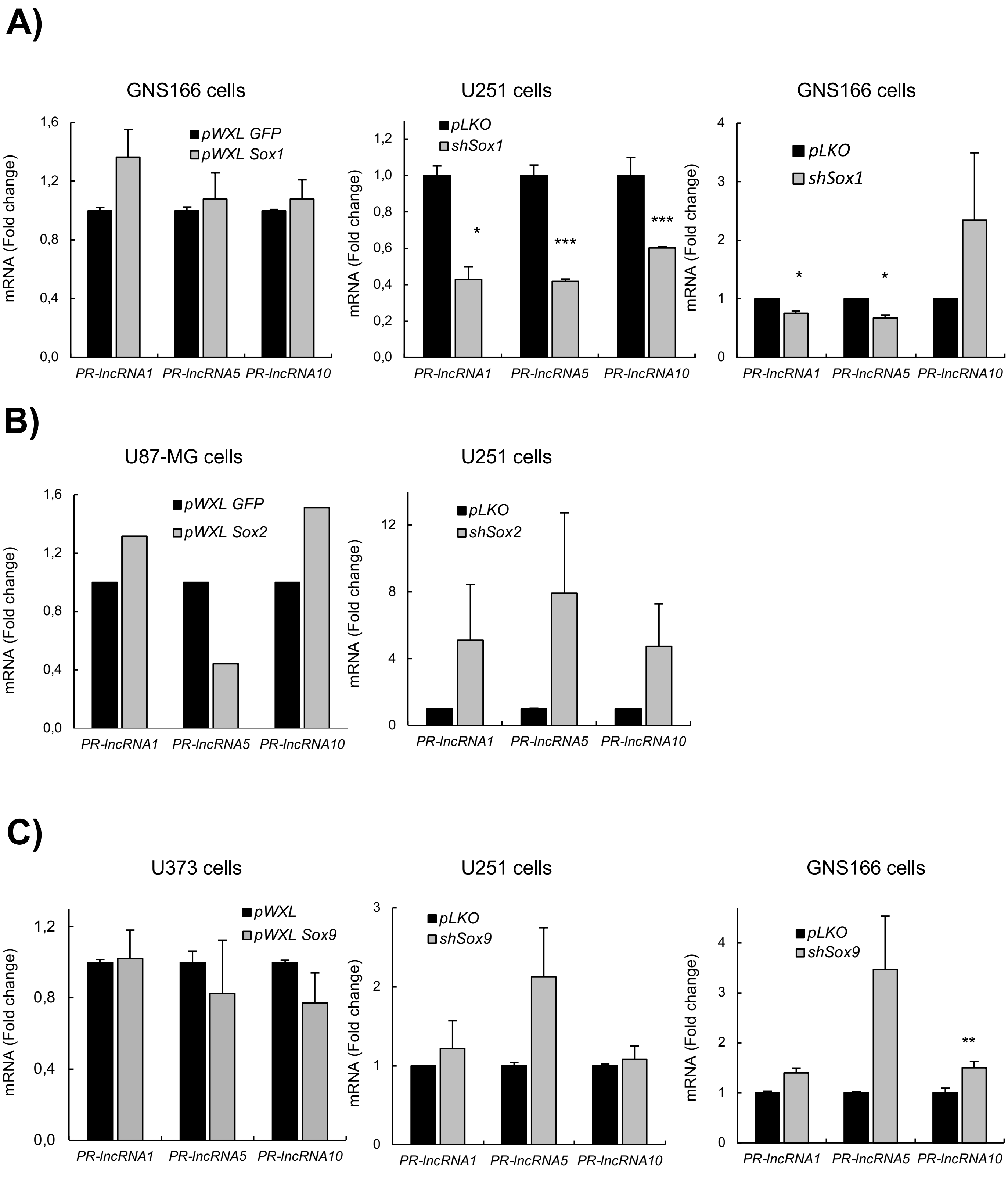
A)
GNS166 cells
pWXL GFP
pWXL Sox1
mRNA (Fold change)
1,6
1,2
0,8
0,4
0,0
PR-lncRNA1
PR-lncRNA5
PR-lncRNA10
U251 cells
pLKO
shSox1
1,2
1,0
0,8
0,6
0,4
0,2
0,0
*
***
***
GNS166 cells
pLKO
shSox1
4
3
2
1
0
B)
U87-MG cells
pWXL GFP
pWXL Sox2
U251 cells
pLKO
shSox2
12
8
4
0
C)
U373 cells
pWXL
pWXL Sox9
U251 cells
pLKO
shSox9
GNS166 cells
pLKO
shSox9
**

Figure Suppl 1. Expression of *PR-LncRNAs* in cells with overexpression or knockdown of *SOX* members

(A) Expression of *PR-LncRNA1, 5* and *10* in GNS166 cells overexpressing *SOX1* and in U251 and GNS166 cells with lentiviral Knock-down of *SOX1*. Data represents the average of 3 independent experiments. Cells were described in (22).

(B) Expression of *PR-LncRNA1, 5* and *10* in U87 cells overexpressing *SOX2* and in U251 cells with lentiviral Knock-down of *SOX2*. Data represents the average of 1 and 2 independent experiments. Cells were described in (21).

(C) Expression of *PR-LncRNA1, 5* and *10* in U373 cells overexpressing *SOX9* and in U251 and GNS166 cells with lentiviral Knock-down of *SOX9*. Data represents the average of 2 and 3 independent experiments. Cells were described in (22) and unpublished results.

P values were determined by Student's *t* test. Asterisks (*,**,***) indicate statistical significance ($p < 0.05$, $p < 0.01$ and $p < 0.005$).
